# Melatonin attenuates hepatic oxidative stress by regulating the P62/LC3 autophagy pathway in PCOS

**DOI:** 10.1530/EC-24-0303

**Published:** 2024-11-21

**Authors:** Junhui Zhang, Hongyan Zhang, Bao Guo, Jun Yang, Renxiang Yu, Wenxiu Chen, Muxin Zhai, Yuhan Cao, Yajing Liu, Qiang Hong, Fenfen Xie

**Affiliations:** 1 Department of Obstetrics and Gynecology, The Second Affiliated Hospital of Anhui Medical University, Hefei, Anhui, China; 2 Department of Histology and Embryology, Anhui Medical University, Hefei, Anhui, China; 3 Second Clinical Medical College, Anhui Medical University, Hefei, Anhui, China; 4 First Clinical Medical College, Anhui Medical University, Hefei, Anhui, China; 5 NHC Key Laboratory of Study on Abnormal Gametes and Reproductive Tract (Anhui Medical University), Hefei, Anhui, China; 6 Key Laboratory of Population Health Across Life Cycle (Anhui Medical University), Ministry of Education of the People’s Republic of China, Hefei, Anhui, China; 7 Department of Obstetrics and Gynecology, The First Affiliated Hospital of Anhui Medical University, Hefei, Anhui, China

**Keywords:** autophagy, melatonin, oxidative stress, polycystic ovary syndrome

## Abstract

The potential protective mechanisms of MT on hepatic OS in PCOS. MT could lead to a decrease in autophagy, presenting a decrease of LC3 and an increase of P62 in the PCOS liver. Increased P62 could reduce Nrf2 ubiquitination and increase its nuclear translocation and activation through direct interaction with Keap1. Thus, MT ultimately activated the Keap1-Nrf2 pathway, promoting nuclear Nrf2 expression to reduce hepatic OS.

**Abstract:**

The elevated level of hepatic oxidative stress (OS) in polycystic ovary syndrome (PCOS) is one of the important causes of liver abnormalities. Therefore, decreasing the level of hepatic OS in PCOS is beneficial in reducing the risk of PCOS-related liver diseases. Melatonin (MT) is recognized as a potent antioxidant. Nevertheless, the efficacy of MT in alleviating hepatic OS associated with PCOS is yet to be established, and the precise mechanisms through which MT exerts its antioxidant effects remain to be fully elucidated. The aim of this study was to explore the potential mechanisms by which MT reduces hepatic OS in PCOS. First, we detected elevated OS levels in PCOS samples. Subsequently, with MT pretreatment, we discovered that MT could significantly diminish the levels of OS, liver triglycerides, total cholesterol, alanine aminotransferase, and aspartate aminotransferase, while concurrently ameliorating mitochondrial structural damage in the PCOS liver. Furthermore, we identified elevated autophagy levels in the liver of PCOS rats and an inhibition of the Keap1–Nrf2 pathway. Through MT pretreatment, the expression of LC3 was significantly decreased, while the Keap1–Nrf2 pathway was activated. Our study showed that MT could affect the Nrf2 pathway dependent on the P62/LC3 autophagy pathway, thereby attenuating hepatic OS in PCOS. These findings offer novel insights and research avenues for the study of PCOS-related liver diseases.

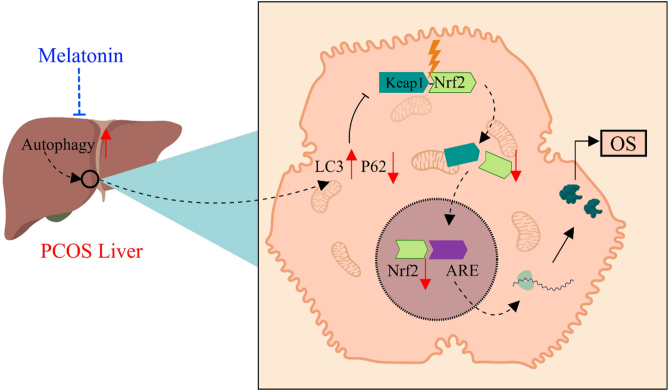

## Introduction

Polycystic ovary syndrome (PCOS) is one of the most common female reproductive endocrine diseases. Recently, the global incidence rate has been as high as 15% (1). PCOS causes significant harm that seriously affects female reproductive and metabolic functions. The main clinical manifestations include menstrual disorders, ovulation disorders, insulin resistance, and hyperandrogenemia. Although many studies have focused on PCOS, the specific cause of PCOS is still unclear (2).

Oxidative stress (OS) is a state characterized by pro-oxidant molecules and antioxidant defense imbalance, including that of reactive oxygen species and nitrogen species. OS participates in the development process of PCOS, and PCOS also promotes OS (3). Studies have shown that abnormal insulin metabolism in PCOS, as evidenced by decreased hepatic insulin extraction as well as abnormal insulin receptor signaling, promotes the development of OS (4). OS is one of the important causes of liver abnormalities in liver-related diseases of PCOS (5). A higher incidence of nonalcoholic fatty liver disease (NAFLD) is often associated with PCOS. OS, insulin resistance, and high androgen levels in PCOS commonly play a key role in the pathogenesis of NAFLD (6). Therefore, the risks of liver diseases related to PCOS might be partly reduced by resisting OS.

Autophagy is a process characterized by the formation of autophagosomes (7). It eliminates discarded proteins and organelles to maintain homeostasis and survival within the intracellular environment, performing an important regulatory function in the occurrence and development of many diseases, including PCOS (8). In addition to the indispensable role of starvation-induced autophagy in liver diseases, basic and selective autophagy also helps to effectively control the quality of organelles and cytoplasmic proteins in hepatocytes (9). Autophagy plays a key role in regulating the physiological and homeostatic aspects of the liver (10). Impairment of autophagic function can lead to the occurrence of various liver diseases (11). Moreover, autophagy also participates in the antioxidant process of the liver.

Autophagy can regulate OS. A recent study showed that uncoordinated 51-like kinase 1 (ULK1) could limit cytotoxicity and hepatic OS induced by saturated fat through autophagy (12). In addition, it activates the nuclear factor-erythroid-2-related factor 2 (Nrf2) antioxidant reaction by degrading Kelch-like ECH-associated protein 1 (Keap1). OS mediated by the Keap1–Nrf2 pathway is the core mechanism for regulating the oxidant–antioxidant balance. Recent research suggests that the autophagy-associated protein P62 can reduce Nrf2 ubiquitination, increase its nuclear translocation and activation, and improve antioxidant stress ability through direct interaction with Keap1 (13).

Melatonin (MT), a lipophilic indole amine hormone, exhibits a circadian secretion pattern originating from the pineal gland (14). Our previous investigations have substantiated its significant therapeutic potential in ameliorating ovarian dysfunction associated with PCOS (15). Notably, MT biosynthesis also occurs in the liver, and its receptors (G protein-coupled receptors MT1A and MT1B) are variably expressed across hepatocytes and influenced by MT levels (16). Moreover, MT can scavenge reactive oxygen species (ROS) and control the endogenous oxidative state of cells by indirectly stimulating certain antioxidant enzymes, such as superoxide dismutase (SOD) (17). MT has been found to ameliorate the development of diet-induced NAFLD by modulating lipid metabolism, attenuating OS, and inhibiting stellate cell activation (18, 19, 20). Given the intimate interplay between MT, autophagy, and OS, it is conceivable that MT may alleviate hepatic OS in PCOS by regulating autophagy.

This study aims to explore whether MT activates the Keap1–Nrf2 pathway through autophagy, thereby reducing the level of hepatic OS in PCOS, eventually paving new avenues and perspectives for investigating liver diseases associated with PCOS.

## Materials and methods

### Ethics authorization

This study received approval from the Medical Ethics Committee of the First Affiliated Hospital of Anhui Medical University. The assigned protocol number for animal subjects was LLSC20170062, while that for human subjects was 20170046. All animal experiments and nursing procedures were carried out in compliance with the animal experimental guidelines of Anhui Medical University.

### Population sample collection

All the participants were 25- to 31-year-old women who received medical treatments at the First Affiliated Hospital of Anhui Medical University from April 2023 to May 2023. Informed consent was obtained from all patients before starting the study. Samples were collected from both the non-PCOS group and the PCOS population, with a standard body mass index (BMI) of 18.5–24.9 kg/m^2^. The diagnostic criteria for PCOS met the Rotterdam Consensus Conference (ESHRE/ASRM 2003). Women who were unable to conceive naturally due to male infertility or tubal disease alone but had normal ovulation cycles were considered part of the non-PCOS group. Each group contained 20 patients who voluntarily underwent *in vitro* fertilization or intracytoplasmic sperm injection to achieve pregnancy. Their sera were collected while they were fasting. The clinical characteristics of the PCOS group and non-PCOS group are presented in Table 1.

**Table 1:** Clinical features of PCOS and non-PCOS group.

Clinical parameter	Non-PCOS (*n* = 20)	PCOS (*n* = 20)
Age (years)	28.50 ± 1.61	27.50 ± 2.01
BMI (kg/m^2^)	20.80 ± 1.78	21.86 ± 1.48
FSH (IU/L)	6.52 ± 1.32	6.03 ± 1.57
LH (IU/L)	4.68 ± 1.55	11.01 ± 7.54^a^
LH/FSH	0.73 ± 0.22	1.79 ± 1.02^a^
T (nmol/L)	0.63 ± 0.29	2.71 ± 0.49^a^
P (nmol/L)	1.43 ± 1.55	3.15 ± 1.76
PRL (ng/mL)	15.41 ± 8.15	15.10 ± 6.51
E2 (pmol/L)	113.70 ± 74.54	163.88 ± 0.05

Serum levels of LH, LH/FSH, and T were significantly higher in the PCOS group compared to the non-PCOS group.

^a^*P* < 0.01 vs non-PCOS.

BMI, body mass index; E2, estradiol; FSH, follicle-stimulating hormone; LH, luteinizing hormone; PCOS, polycystic ovary syndrome; P, progesterone; PRL, prolactin; T, testosterone.

### PCOS rat model

From Beijing Vital River Laboratory Animal Technology Co., Ltd., 60 female Sprague‒Dawley rats (25 days old) were purchased. They were housed in a standard specific pathogen-free animal laboratory. For 3–5 days, the rats were subjected to a 12-h light:12-h darkness cycle at a room temperature of 20–24°C and humidity of 60–65%. After 3 days of acclimation, the rats were randomly divided into four groups: the control group, the MT group, DHEA group, and DHEA + MT group. In the control group, saline (1 mL/100 g body weight) was administered intragastrically at 08:30 h, followed by a subcutaneous injection of corn oil (0.1 mL/100 g body weight, Sigma) at 09:30 h. For the MT group, MT (5 mg/100 g body weight, Sigma) was administered intragastrically at 08:30 h, with a corn oil injection at 09:30 h. In the DHEA group, saline was administered intragastrically at 08:30 h, and DHEA (6 mg/100 g body weight, MCE) dissolved in corn oil was injected at 09:30 h. Finally, in the DHEA + MT group, MT was administered intragastrically at 08:30 h, followed by a subcutaneous injection of DHEA at 09:30 h. This protocol was followed for 20 consecutive days.

### Vaginal smears

Starting from the 10th day of PCOS modeling, vaginal secretions from the rats were collected and observed under an optical microscope between 08:00 h and 09:00 h. Three types of cells were identified. Cells with a round shape possessing nuclei were classified as epithelial cells; cells with an irregular shape lacking nuclei were termed keratinized cells; and small round cells were identified as white blood cells. The stages of the estrous cycle were determined based on the ratios among these cells.

### Cell culture and treatment

HepG2 cells were cultured in high glucose DMEM with the addition of 10% fetal bovine serum (Gibco), 0.1 mg/mL streptomycin, and 100 units/mL penicillin G (Beyotime). The cells were seeded into a six-well plate at a density of 2.0 × 10^5^ cells per well. Subsequently, the cells were treated for 48 h with H_2_O_2_ (400 µΜ, Sigma), MT (200 µΜ, Sigma), rapamycin (50 nM, MCE), ML385 (5 µM, MCE), and TBHQ (50 µM, MCE), either individually or in combination.

### Hematoxylin-eosin staining

The liver and ovarian sections of the rats were dewaxed using xylene and then stained successively with hematoxylin and eosin dyes. The slices were then dehydrated, made transparent, and sealed. Images were collected and analyzed using a 3D digital slicing scanner (Pannoramic MIDI, 3DHISTECH).

### Real-time qPCR analysis

Total RNA from rat liver was extracted with HyperPure RNA Isolation Kit (EnzyArtisan). The concentration of total RNA was assessed through absorbance spectroscopy (Nanodrop ND-2000, Thermo Fisher). This was followed by the conversion of RNA to cDNA with HyperScript III RT SuperMix (EnzyArtisan). Real-time qPCR analysis was performed using 2× SYBR Green I Master (Roche Diagnostics GmbH) with the LightCycler 480 real-time PCR system. All primers were synthesized and purified by EnzyArtisan. The primer sequences were as follows: beta-actin: GACGTTGACATCCGTAAAGACC (F), CTAGGAGCCAGGGCAGTAATCT (R); MT1A: GGATGGAACCTGGGATATCTGC (F), GTTGAATACCGAGCCAATGACAC (R); MT1B: ACCTGCGAATATGGATACTGGTG (F), CCACAAACACTGCGAACATGG (R). All qPCRs were carried out in a final volume of 20 μL.

### Immunohistochemical staining

Paraffin sections of rat liver were dewaxed and dehydrated, followed by antigen retrieval, endogenous peroxidase blocking, and primary antibody application. The specific primary antibodies used in immunohistochemistry (IHC) were as follows: Keap1 (1:100, rabbit monoclonal; Affinity, #AF5266), Nrf2 (1:100, rabbit monoclonal; Affinity, #AF0639), HO-1 (1:200, rabbit monoclonal; Proteintech, 10701-1-AP), SOD1 (1:800, rabbit monoclonal; Proteintech, 10269-1-AP), and SOD2 (1:1000, rabbit monoclonal; Proteintech, 24127-1-AP). Next, the enzyme-labeled goat anti-mouse/rabbit IgG polymer was added, and the slices were then dehydrated with alcohol, made transparent with xylene, and finally sealed with neutral gum. Images were collected and analyzed using a 3D digital slicing scanner (Pannoramic MIDI, 3DHISTECH).

### Immunofluorescence staining

To detect the levels of OS and autophagy in the liver and HepG2 cells, liver paraffin sections and HepG2 cells were used to detect HO-1 (1:200, rabbit monoclonal; Proteintech, 10701-1-AP), SOD1 (1:50, rabbit monoclonal; Proteintech, 10269-1-AP), SOD2 (1:100, rabbit monoclonal; Proteintech, 24127-1-AP), P62 (1:100, mouse monoclonal; Abcam, ab56416), and LC3B (1:100, rabbit monoclonal; Sigma, L7543) indicators. They were then incubated overnight at 4°C. Following this, a thorough washing was performed three times, and the sections were subsequently incubated with a goat anti-mouse/rabbit IgG secondary antibody (1:250, Immunoway, RS3608, RS0004) for 60 min at 37°C. DAPI was utilized to stain the nuclei, and the cells were washed three additional times with PBS. Finally, the slides were incubated with an anti-fluorescence quencher. The stained cells were observed under a fluorescence microscope (Thunder Image, 3D Assay).

### Detection of apoptosis by TUNEL assay

First, the liver paraffin sections are subjected to a dewaxing treatment. Then, the permeability of the sample is increased. Next, the labeling reaction solution (AT005, 7seabiotech) is prepared, and the labeling reaction is performed. Finally, the reaction is terminated, and the results are evaluated. The PI nuclear stain can color the DNA of all labeled and unlabelled cells red, with only the apoptotic cells containing FITC-dUTP incorporation and localization showing green fluorescence.

### Transmission electron microscopy

Several pieces of 1 mm^3^ liver tissue were obtained and immediately fixed in phosphate buffer containing 2.5% glutaraldehyde for 12 h. After that, they were fixed with 1% osmic acid for 1–2 h. These tissues were dehydrated using a gradient ethanol series and embedded in epoxy resin. The resin-coated blocks were then cut into 70 nm ultrathin slices using an ultrathin microtome (UC-7, Leica). Images of the ultrathin sections were taken using a transmission electron microscope (TEM) (JEM1400Flash) while they were mounted on a copper mesh.

### Biochemical analysis

The contents of malondialdehyde (MDA) in the samples were determined using a thiobarbituric acid detection kit. A multifunctional enzyme labeler (MD/SpectraMAX ID3) was used to measure the optical density at 532 nm. The activity of glutathione peroxidase (GSH-PX) was expressed in terms of the rate of its enzymatic reaction. Catalase (CAT) and SOD activities were respectively determined by the ammonium molybdate method and the WST-1 method. To calculate the oxidized glutathione (GSSG)/glutathione (GSH) ratio, total GSH and its oxidized form (GSSG) were spectrophotometrically evaluated by monitoring the change in absorbance at 405 nm. Liver triglyceride (TG) and total cholesterol (TC) levels were respectively measured by the GPO-PAP enzymatic method and the COD-PAP method. Alanine aminotransferase (ALT) and aspartate aminotransferase (AST) levels were measured using the Reitman–Frankel method to assess liver function. The optical density value was respectively measured at 505 nm and 510 nm according to the instructions of the test kits (Nanjing Institute of Jiancheng Bioengineering).

### Western blotting

The specific methods for protein extraction from rat liver tissue and HepG2 cells were described in previous studies (15). The protein samples were added to a 10% or 12% SDS-PAGE gel for electrophoresis and then transferred onto a PVDF membrane. After that, the PVDF membrane was incubated with primary antibody overnight. The primary antibodies used were as follows: LC3B (1:1000, rabbit monoclonal; Sigma, L7543), Beclin1 (1:1000, rabbit monoclonal; Cell Signaling Technology, D40C5), P62 (1:1000, mouse monoclonal; Abcam, ab56416), Nrf2 (1:1000, rabbit monoclonal; Affinity, #AF0639), Keap1 (1:500, rabbit monoclonal; Affinity, #AF5266), HO-1 (1:1000, rabbit monoclonal; Proteintech, 10701-1-AP), SOD1 (1:5000, rabbit monoclonal; Proteintech, 10269-1-AP), SOD2 (1:5000, rabbit monoclonal; Proteintech, 24127-1-AP), GAPDH (1:5000, mouse monoclonal; Immunoway, YM3029), and Lamin B1 (1:5000, rabbit monoclonal; Abcam, ab16048). The PVDF membrane was then washed and incubated with a horseradish peroxidase–labeled secondary antibody (1:10,000, Bioworld, BS12478, BS13278) at room temperature for 1 h. The target bands were observed using ECL chemiluminescence substrate (Biosharp) and quantified using ImageJ software (version 1.46). GAPDH and Lamin B1 were used as internal parameters to calculate protein expression.

### Statistical analysis

The results are presented as the mean ± s.d. for continuous variables. Statistical data were determined by *t*-test and one-way ANOVA using SPSS 17.0 software. A *P* value <0.05 was considered statistically significant. All experiments were replicated three times.

## Results

### The increased OS levels of serum in the human PCOS group

**Figure 1:**
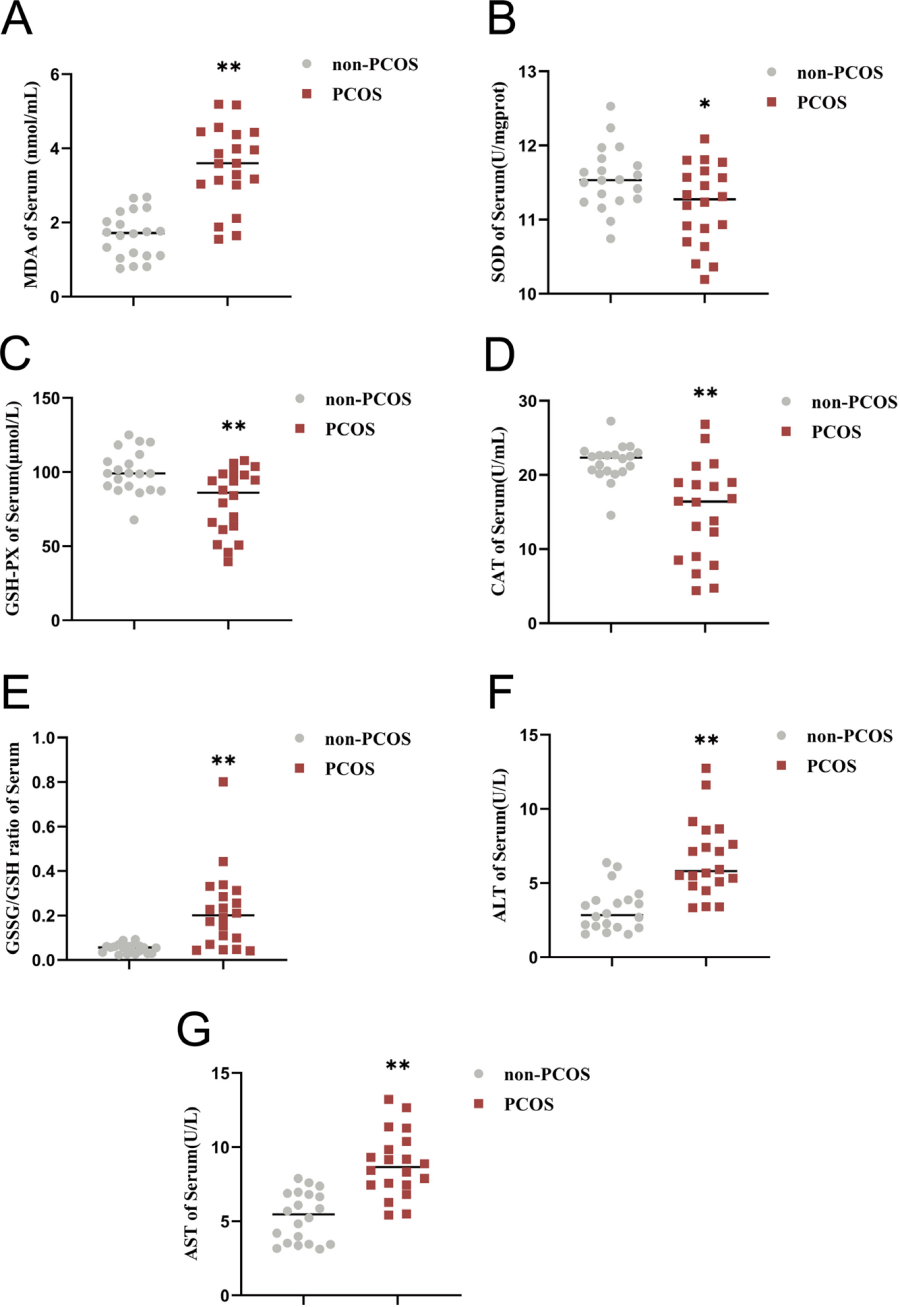
Increased levels of OS in the serum of the human PCOS group.(A–E)Changes in the contents of MDA, antioxidant enzymes SOD, GSH-PX, CAT, and the GSSG/GSH ratio in the serum of humans in two groups (*n* = 20). (F–G) ALT and AST levels in serum in two groups (*n* = 20). Data were presented as the mean ± s.d. *P < 0.05 vs control; **P < 0.01 vs control.

To investigate the changes in OS in PCOS patients, we used the biochemical process to assess the concentration of the lipid peroxidation product MDA, the GSSG/GSH ratio, and antioxidant enzymes SOD, GSH-PX, and CAT in serum samples. Significantly elevated levels of MDA and the GSSG/GSH ratio, along with decreased levels of SOD, GSH-PX, and CAT, were found in PCOS patients (Fig. 1A, B, C, D, and E). Subsequent measurement of serum levels of ALT and AST found significantly higher activities in the PCOS group (Fig. 1F and G). This finding suggested a potential link between increased OS levels and liver function abnormalities in PCOS.

### MT reduced OS in the livers of PCOS rats

**Figure 2:**
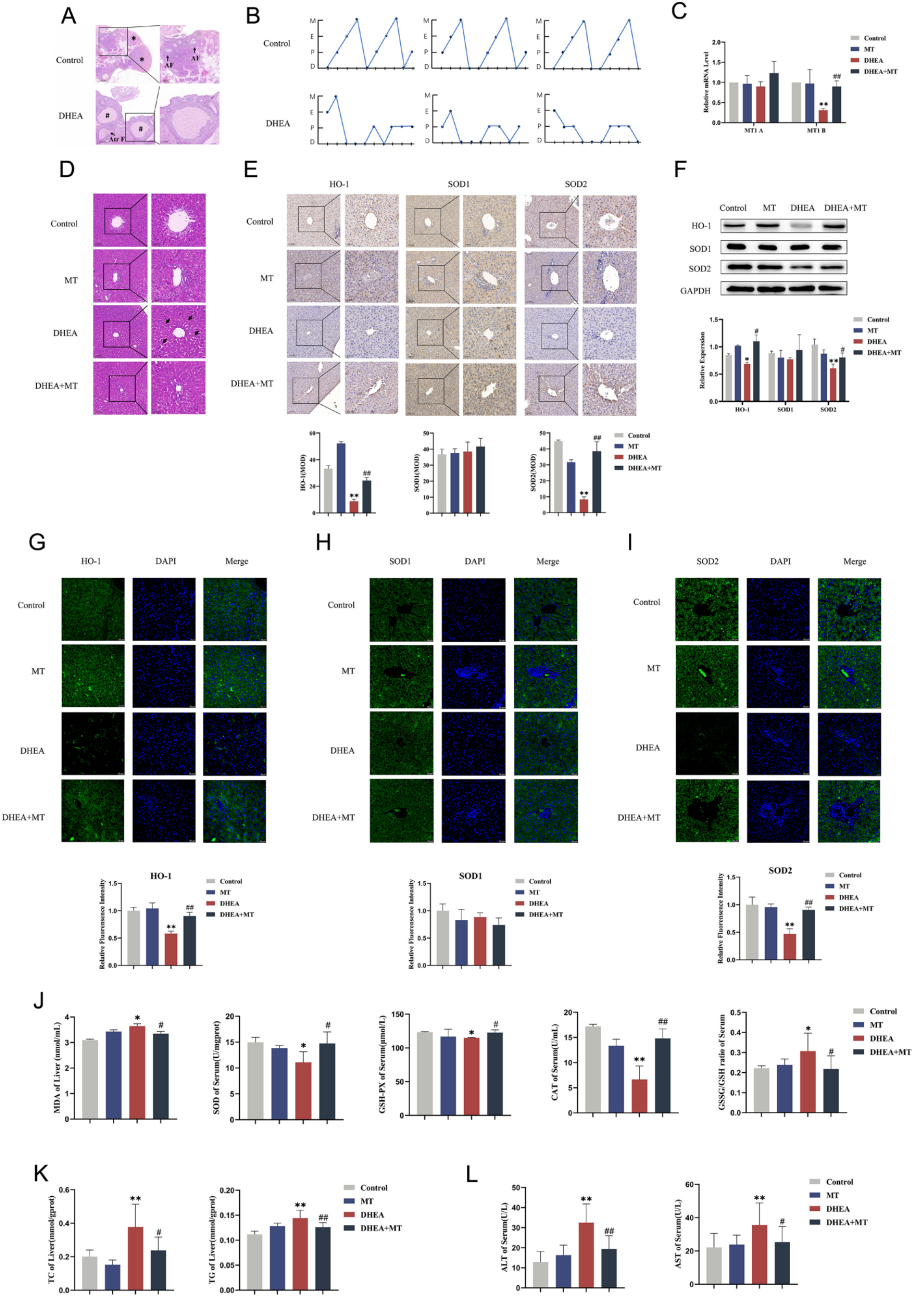
MT attenuated hepatic OS in PCOS rats. The rats were randomly divided into four groups: control group, MT group, DHEA group, and DHEA + MT group. (A) Morphological changes of ovaries in the two groups with HE staining. Scale bar: 200 μm. *, corpus luteum; #, cystic follicle; AF, antral follicles; Atr F, atretic follicles (*n* = 3).(B) Estrus cyclicities in two groups (*n* = 3). (C) Relative mRNA levels of MT1A and MT1B in rat livers of the four groups (*n* = 3). (D) Morphological structures of rat livers in the four groups with HE staining. →: hepatic cords. Scale bar: 50 μm (*n* = 3). (E)Immunohistochemistry analysis of HO-1, SOD1, and SOD2 in rat livers of the four groups. Scale bar: 50 μm (*n* = 3). (F)Western blot analysis of HO-1, SOD1, and SOD2 expressions in rat livers of four groups (*n* = 3). (G–I)Immunofluorescent staining of HO-1, SOD1, and SOD2 in rat livers of the four groups. Scale bar: 20 μm (*n* = 3). (J) Changes in the contents of MDA and antioxidant enzymes SOD, GSH-PX, CAT, and the GSSG/GSH ratio in rat livers and serum of the four groups (*n* = 5). (K) Changes in the contents of TC and TG in rat livers of the four groups (*n* = 5). (L) ALT and AST levels in rat serum of the four groups (*n* = 5). Data were presented as the mean ± s.d. *P < 0.05 vs control; **P < 0.01 vs control; ^#^*P* < 0.05 vs DHEA; ^##^*P* < 0.01 vs DHEA.

To explore OS in the PCOS liver, we used DHEA to establish a PCOS rat model. We observed typical morphological changes resembling polycystic ovarian characteristics (Fig. 2A) and irregular estrus cyclicity (Fig. 2B). Histological analysis of the ovarian follicles revealed that the DHEA group had fewer corpus luteum and antral follicles, alongside increased atretic follicles, compared to the control group (Fig. 2A). The pathological results showed that the hepatic cords in the hepatic tissues of mice in the DHEA group were irregular (Fig. 2D). There was no statistically significant difference in MT1A mRNA levels; however, MT was able to restore the DHEA-decreased MT1B levels (Fig. 2C). Next, we continued to analyze the effects of MT on hepatic OS in PCOS. IHC, western blotting, immunofluorescence, and biochemical processes were applied to detect OS-related indicators in the liver tissues and serum. Specifically, we observed a decrease in the expression levels of HO-1 and SOD2 in the DHEA group. In alignment with clinical samples, the DHEA group exhibited significantly elevated MDA concentrations and a higher GSSG/GSH ratio, coupled with decreased levels of SOD, GSH-PX, and CAT levels, thereby recapitulating the observed profile of OS in PCOS patients. However, the expression levels of SOD, GSH-PX, and CAT were significantly increased in the DHEA + MT group (Fig. 2E, F, G, H, I, and J), showing that MT might alleviate hepatic OS in PCOS rats. Finally, we demonstrated that MT treatment effectively alleviated hepatic lipid abnormalities and subsequently restored liver function, as evidenced by a reduction in TC, TG, ALT, and AST concentrations (Fig. 2K and L).

### MT ameliorated hepatic mitochondrial structural damage in PCOS rats

**Figure 3:**
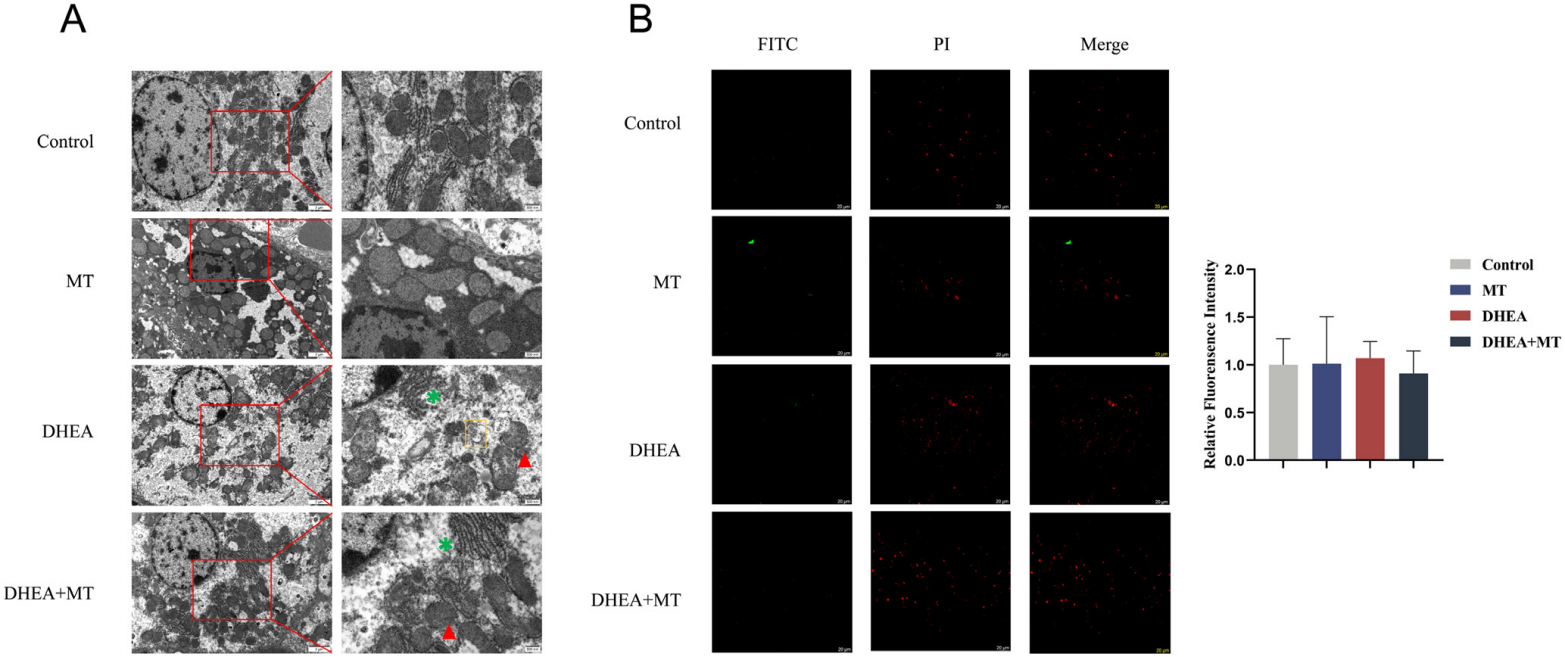
MT attenuated hepatic mitochondrial damage in PCOS rats. (A)TEM examination of rat livers in the four groups. Scale bar: 500 nm. ▲, mitochondria; *, rough endoplasmic reticulum; ◻, autophagosome (*n* = 3).(B)Immunofluorescent staining showing TUNEL-stained cells in rat livers of the four groups. Scale bar: 20 μm (*n* = 3).

TEM was used to examine the ultrastructural changes in rat liver tissues. As shown in Fig. 3A, the DHEA group exhibited a loss of mitochondrial quantity, mitochondrial membrane rupture, blurred mitochondrial cristae, twisted and fractured rough endoplasmic reticulum, and increased autophagosomes. Conversely, the ultrastructure and quantity of the mitochondria were restored, and the fracture of the rough endoplasmic reticulum was improved significantly with pretreatment with MT. There was no significant difference in TUNEL positivity observed in the immunofluorescence staining of liver sections from rats (Fig. 3B).

### MT attenuated hepatic OS by inhibiting autophagy in PCOS

**Figure 4:**
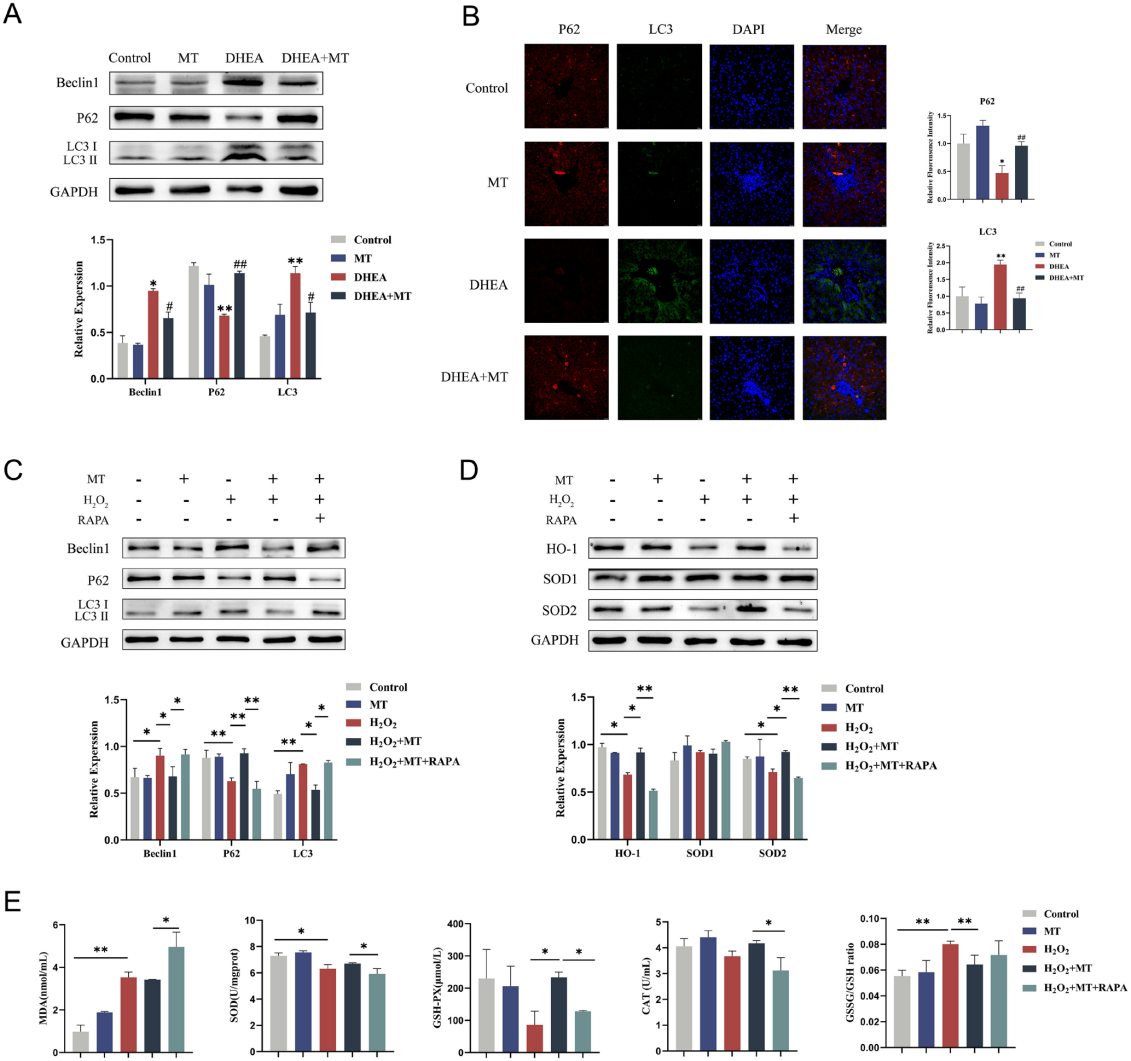
MT inhibited autophagy to reduce hepatic OS in PCOS. (A) Western blot analysis of Beclin1, P62, and LC3 expressions in rat livers of four groups (*n* = 3). (B) Immunofluorescent staining of P62 and LC3 in rat livers of four groups. Scale bar: 20 μm (*n* = 3). (C) Western blot analysis of Beclin1, P62, and LC3 expressions in HepG2 cells of five groups (*n* = 3).(D)Western blot analysis of HO-1, SOD1, and SOD2 expressions in HepG2 cells of five groups (*n* = 3).(E)Changes in the contents of MDA and antioxidant enzymes SOD, GSH-PX, and CAT in HepG2 cell supernatants of five groups. The GSSG/GSH ratio in HepG2 cells of five groups (*n* = 5). Data were presented as the mean ± s.d. *P < 0.05; **P < 0.01; ^#^*P* < 0.05; ^##^*P* < 0.01.

Autophagy is closely related to OS (21, 22). To investigate the effect of MT on autophagy and its role in regulating hepatic OS in PCOS, western blotting was conducted to measure the expression levels of autophagy. Compared to the control group, the DHEA group exhibited an increased level of autophagy in the liver. Additionally, the expression levels of Beclin1 and LC3 II were increased, while the expression level of P62 was decreased (Fig. 4A and B). TEM photographs of liver tissues also showed an increase in autophagosomes (Fig. 3A). However, the levels of autophagy were decreased in the DHEA + MT group (Fig. 4A and B). Consistent results were obtained from HepG2 cells in both the H_2_O_2_-exposed and H_2_O_2_ + MT groups (Fig. 4C). To further explore the relationship between autophagy and OS, we added the autophagy activator rapamycin to HepG2 cells co-treated with H_2_O_2_ + MT. The levels of autophagy and OS in HepG2 cells treated with rapamycin were higher than those in the H_2_O_2_ + MT group (Fig. 4C and D). Subsequently, the level of MDA was also higher in the supernatants of the H_2_O_2_ + MT + RAPA group than in the H_2_O_2_ + MT group. The ratio of GSSG/GSH was elevated in both the H_2_O_2_ and the H_2_O_2_ + MT + RAPA groups, whereas it was reduced in the H_2_O_2_ + MT group, indicating that the protective function of MT against OS was inhibited (Fig. 4E). These results showed that pretreatment with MT could inhibit autophagy to reduce OS in PCOS livers.

### MT alleviated hepatic OS by activating the Nrf2 pathway depended on the P62 autophagy pathway in PCOS

**Figure 5:**
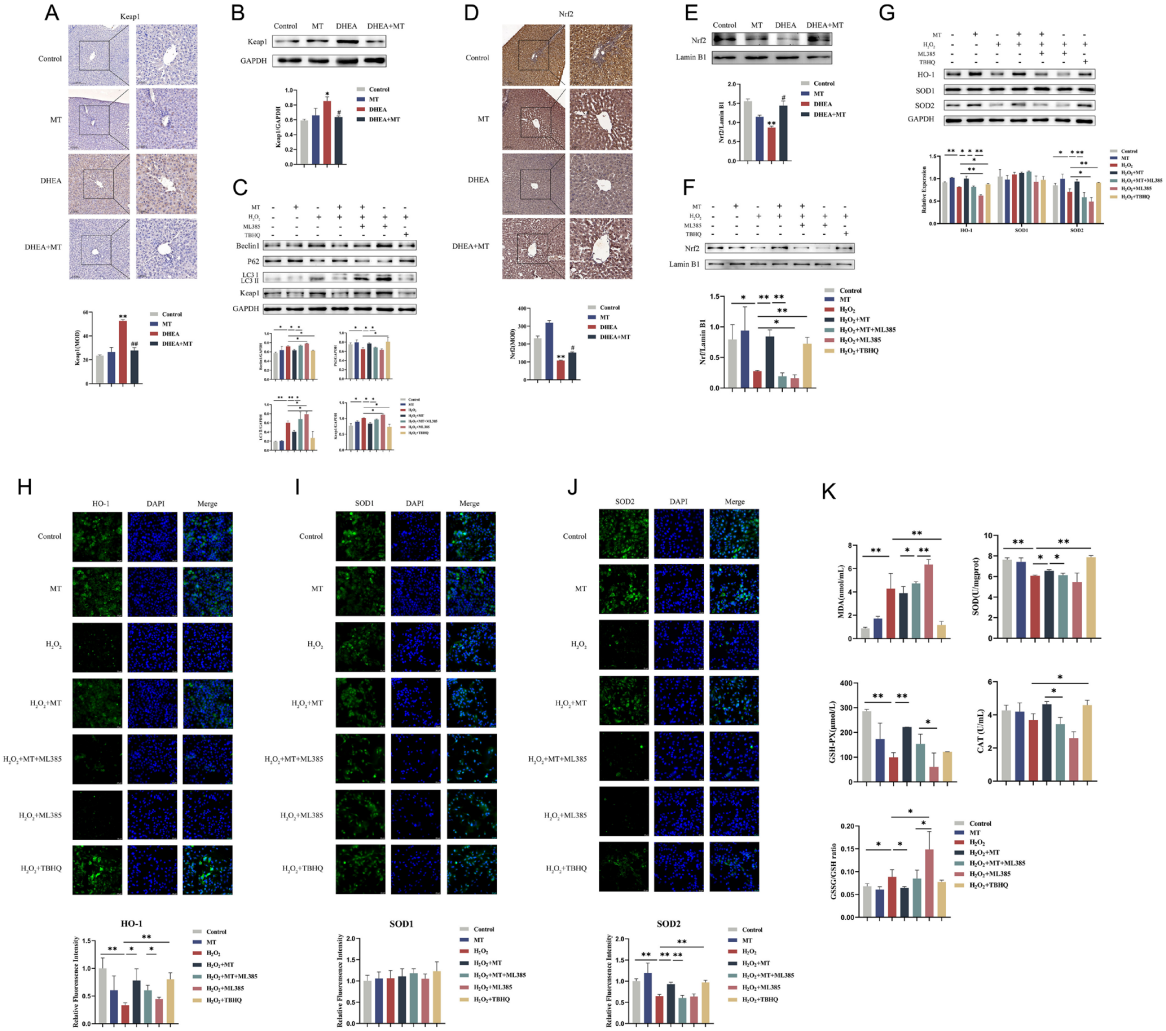
MT regulated the Nrf2 pathway to attenuate hepatic OS via the P62 autophagy pathway in PCOS. (A) Immunohistochemistry analysis of Keap1 in rat livers of four groups. Scale bar: 50 μm (*n* = 3). (B)Western blot analysis of Keap1 expression in rat livers of four groups (*n* = 3).(C) Western blot analysis of Beclin1, P62, LC3, and Keap1 expressions in HepG2 cells of seven groups (*n* = 3). (D)Immunohistochemistry analysis of Nrf2 in rat livers of four groups. Scale bar: 50 μm (*n* = 3). (E) Western blot analysis of Nrf2 expression in rat livers of four groups (*n* = 3). (F) Western blot analysis of Nrf2 expression in HepG2 cells of seven groups (*n* = 3). (G)Western blot analysis of HO-1, SOD1, and SOD2 expressions in HepG2 cells of seven groups (*n* = 3). (H–J)Immunofluorescent staining of HO-1, SOD1, and SOD2 in HepG2 cells of seven groups. Scale bar: 20 μm (*n* = 3).(K)Changes in the contents of MDA and antioxidant enzymes SOD, GSH-PX, and CAT in HepG2 cell supernatants of seven groups. The GSSG/GSH ratio in HepG2 cells of seven groups (*n* = 5). Data were presented as the mean ± s.d. *P < 0.05; **P < 0.01; ^#^*P* < 0.05; ^##^*P* < 0.01.

The Keap1–Nrf2 signaling pathway plays a critical role in the response to antioxidant stress regulated by the P62 autophagy pathway. To speculate whether MT affected the Nrf2 pathway by modulating the P62 autophagy pathway to ameliorate hepatic OS in PCOS, we examined the expression of cytoplasmic Keap1 and nuclear Nrf2. P62 and Nrf2 expression levels were lower, while Keap1 expression levels were higher in the DHEA group than in the control group. However, these changes were significantly reversed in the DHEA + MT group (Figs 4AB, 5A, B, D, andE). Similarly, the results from the cell experiments coincided with those from the animal experiments. After using ML385, a specific Nrf2 inhibitor, the expression of Keap1 was upregulated, while the expressions of Nrf2, HO-1, and SOD2 were downregulated in the H_2_O_2_ and H_2_O_2_ + ML385 groups. However, after using the specific Nrf2 activator TBHQ, the expression of Keap1 was downregulated, and the expressions of Nrf2, HO-1, and SOD2 were upregulated in the H_2_O_2_ + MT and H_2_O_2_ + TBHQ groups (Fig. 5C, F, G, H, I, and J). Moreover, the levels of MDA and the ratio of GSSG/GSH were increased in the H_2_O_2_ and H_2_O_2_ + ML385 groups, contrary to the results from the H_2_O_2_ + MT and H_2_O_2_ + TBHQ groups (Fig. 5K). These findings suggested that MT inhibited autophagy, thereby activating the Nrf2 signaling pathway to exert antioxidant effects.

## Discussion

MT serves as a natural antioxidant, capable of mitigating cellular oxidative damage by alleviating OS (23). Our research revealed that within the liver of PCOS rats, there was no statistically significant difference in MT1A mRNA levels, but MT still managed to replenish the MT1B levels that had been diminished by DHEA. Moreover, through *in vivo* experiments, we have validated that MT could assuage DHEA-induced OS and reduce TC, TG, ALT, and AST levels in the PCOS rat livers. Finally, *in vitro* experiments also elucidated that the antioxidant mechanism mediated by MT dependent on the regulation of the Keap1–Nrf2 pathway via autophagy.

As a cell signaling molecule, ROS are also regarded as toxic byproducts generated during aerobic metabolism (24). Research has shown that excess ROS can lead to OS, resulting in lipid peroxidation, protein distortion, and DNA breakage, ultimately harming hepatocytes (25). TEM results also showed hepatic abnormalities, such as ruptured mitochondrial membranes, blurred mitochondrial cristae, and twisted and fractured rough endoplasmic reticulum, in DHEA-induced PCOS rats. MDA is a natural byproduct of lipid peroxidation in organisms, and the antioxidant enzymes include SOD, CAT, GSH-PX, etc. Lipid peroxidation and decreased levels of antioxidant enzymes are accompanied by OS (26). The ratio between GSSG and GSH has been used as an important *in vitro* and *in vivo* biomarker of the redox balance in cell and, consequently, of cellular OS (27). Our study also indicated that the levels of OS were elevated in the serum of PCOS patients, as well as in the serum and livers of PCOS rats.

The liver is the primary organ tasked with overseeing metabolism and detoxification processes (28). Nonetheless, our current comprehension of the mechanisms that regulate OS within the liver of individuals affected by PCOS remains somewhat limited. In our research, we conducted a comparative analysis of the levels of OS and antioxidant stress indicators among various groups of model animals. The findings from our study emphatically revealed that MT effectively mitigated OS in the livers of PCOS rats. Moreover, it replenished the quantity and enhanced the structure of mitochondria within liver cells, thereby suggesting that MT could play a pivotal role in modulating OS in these cellular entities. Furthermore, Zhang *et al.* discovered that MT improved hepatic inflammation, OS, and mitochondrial autophagy after exposure to ochratoxin A (29). Reports have indicated that MT confers protection to the testes from palmitic acid-induced lipotoxicity through the activation of SIRT1, which alleviates OS (30). Despite these insights, the precise regulatory mechanisms through which MT exerts its influence on hepatic OS continue to be explored.

Autophagy regulates cell metabolism in response to environmental stimuli. However, excessive or insufficient autophagy can lead to diseases (31). Research on arsenic-induced hypertension suggested that MT may exert a protective effect against arsenic-induced vascular toxicity by suppressing apoptosis and modulating the Sirt1/autophagy pathway (32). Scientists have revealed that inhibiting autophagy could limit the cellular toxicity and OS induced by saturated fatty acids in hepatocytes (33). In this study, we observed increased expression of LC3 and Beclin1 in PCOS livers *in vivo* and *in vitro* and decreased expression of P62, which could be reversed by MT. Cell experiments further demonstrated a positive correlation between autophagy and OS. In summary, we believe that MT inhibits autophagy and ultimately suppresses hepatic OS in PCOS. However, there are currently no reports on how autophagy participates in the regulation of hepatic OS in PCOS.

P62 is a protein that binds to ubiquitin and serves as an autophagy receptor, connecting Nrf2 and the autophagy pathway (13, 34). The Keap1–Nrf2 pathway is the primary defense system in cells, safeguarding them from damage caused by OS (35). When P62 is phosphorylated or accumulates abnormally, it binds more strongly to Keap1, leading to the dissociation of Keap1 and Nrf2. As a result, Nrf2 moves to the nucleus and activates downstream regulatory pathways that shield cells from oxidative damage (21, 36). Research has also shown that knockout of Nrf2 reduces its transfer to the nucleus, thereby aggravating inflammation and autophagy. This process mainly depends on the P62–Keap1–Nrf2 signaling pathway, which is a well-known pathway involved in regulating autophagy in response to OS (37). Cheng *et al.* found that MT elicited Nrf2 by disrupting the interaction between Keap1 and Nrf2 to promote antioxidant enzyme expression such as HO-1, which would salvage HCPT-induced ROS production (38). In acute pancreatitis models, the antioxidant sitagliptin inhibits inflammation and ameliorates OS by activating the P62–Keap1–Nrf2 pathway, protecting pancreatic function (39). Our study found that the expression of P62 was significantly decreased in the livers of PCOS rats and in HepG2 cells exposed to H_2_O_2_. Using Nrf2 inhibitors, we demonstrated that inhibiting the Keap1/Nrf2 signaling pathway worsens OS in hepatocytes. Overall, these findings further support that MT improves hepatic OS levels by activating the Keap1–Nrf2 signaling pathway, which is regulated by P62 autophagy.

Mechanisms for improving OS in a damaged liver by antioxidants have been extensively reported (40). These studies have investigated various factors, including autophagy, inflammation, and small RNA regulation (41). However, none of these studies have explored the involvement of MT in regulating hepatic OS in PCOS. Therefore, it is necessary to further elucidate the regulatory mechanisms by which MT affects OS in animal models. In brief, our research first demonstrated that MT plays a protective role in DHEA-induced hepatic OS in PCOS through the P62–Keap1–Nrf2 pathway.

In summary, our study indicated that MT could inhibit autophagy by activating the Keap1–Nrf2 signaling pathway, resulting in reduced hepatic OS in PCOS.

## Declaration of interest

The authors declare that there is no conflict of interest that could be perceived as prejudicing the impartiality of the research reported.

## Funding

The present work was funded by the National Natural Science Foundation Youth Project of China (82101716, 82201804), the Doctoral Research Fund of Anhui Medical Universityhttp://dx.doi.org/10.13039/501100002947 (XJ202002), the Open Project of Anhui Province Key Laboratory of Reproductive Health and Genetics (RHG-2020-8), the Research Fund of Anhui Institute of Translational Medicine (ZHYX2020A001), the National Innovation and Entrepreneurship Training Program for College Students (202310366030), and the Provincial College Students Innovation and Entrepreneurship Training Program (S202210366059, S202310366002X).

## Data availability statement

Raw data and modeling scripts are available on request.

## Author contributions statement

FFX, designed this work; FFX and QH, editing of the study; YJL, technical and material support; JHZ, conducted the *in vivo* experiments; HYZ and BG, performed the* in vitro* experiments; JY, RXY, and MXZ, collected the samples; WXC and YHC, analyzed the data; JHZ, HYZ, BG, and JY, writing the first draft of the manuscript. All the authors have read and approved the final manuscript.
