# ERRATUM: Melatonin attenuates hepatic oxidative stress by regulating the P62/LC3 autophagy pathway in PCOS

**DOI:** 10.1530/EC-24-0303e

**Published:** 2025-02-26

**Authors:** Junhui Zhang, Hongyan Zhang, Bao Guo, Jun Yang, Renxiang Yu, Wenxiu Chen, Muxin Zhai, Yuhan Cao, Yajing Liu, Qiang Hong, Fenfen Xie

**Affiliations:** ^1^ Department of Obstetrics and Gynecology, The Second Affiliated Hospital of Anhui Medical University, Hefei, Anhui, China; ^2^ Department of Histology and Embryology, Anhui Medical University, Hefei, Anhui, China; ^3^ Second Clinical Medical College, Anhui Medical University, Hefei, Anhui, China; ^4^ First Clinical Medical College, Anhui Medical University, Hefei, Anhui, China; ^5^ NHC Key Laboratory of Study on Abnormal Gametes and Reproductive Tract (Anhui Medical University), Hefei, Anhui, China; ^6^ Key Laboratory of Population Health Across Life Cycle (Anhui Medical University), Ministry of Education of the People’s Republic of China, Hefei, Anhui, China; ^7^ Department of Obstetrics and Gynecology, The First Affiliated Hospital of Anhui Medical University, Hefei, Anhui, China

The authors and journal apologise for an error in the above paper, which appeared in volume 13 part 12, article ID e240303. The error relates to Fig. 5J, given on page 10, in which incorrect immunofluorescent staining images were unintentionally included in the H_2_O_2_+ML385 group panels.

The authors state that both HO-1 (Fig. 5H) and SOD2 (Fig. 5J) are indicators of antioxidant capacity and both had decreased expression in the H_2_O_2_+ML385 group in this study. Therefore, this error has no impact on the final conclusions and scientific validity of the article.

The corrected Fig. 5 is given in full below:

**Figure 5:**
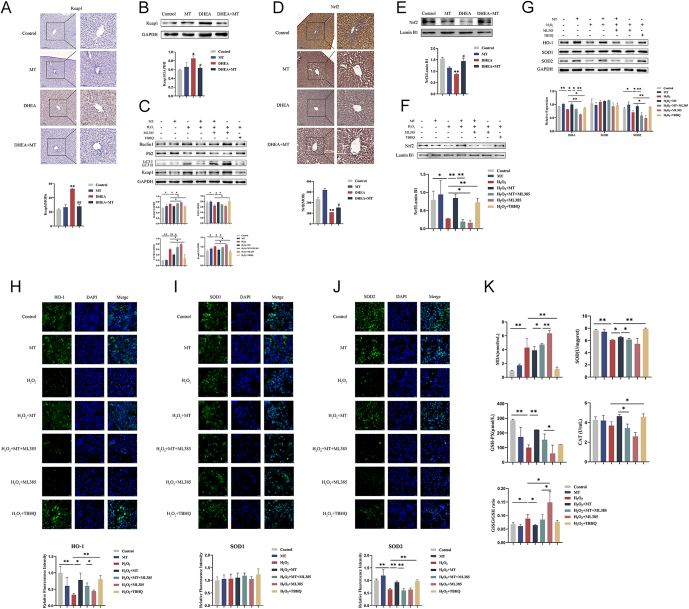
MT regulated the Nrf2 pathway to attenuate hepatic OS via the P62 autophagy pathway in PCOS. (A) Immunohistochemistry analysis of Keap1 in rat livers of four groups. Scale bar: 50 μm (n = 3). (B) Western blot analysis of Keap1 expression in rat livers of four groups (n = 3). (C) Western blot analysis of Beclin1, P62, LC3, and Keap1 expressions in HepG2 cells of seven groups (n = 3). (D) Immunohistochemistry analysis of Nrf2 in rat livers of four groups. Scale bar: 50 μm (n = 3). (E) Western blot analysis of Nrf2 expression in rat livers of four groups (n = 3). (F) Western blot analysis of Nrf2 expression in HepG2 cells of seven groups (n = 3). (G) Western blot analysis of HO-1, SOD1, and SOD2 expressions in HepG2 cells of seven groups (n = 3). (H–J) Immunofluorescent staining of HO-1, SOD1, and SOD2 in HepG2 cells of seven groups. Scale bar: 20 μm (n = 3). (K) Changes in the contents of MDA and antioxidant enzymes SOD, GSH-PX, and CAT in HepG2 cell supernatants of seven groups. The GSSG/GSH ratio in HepG2 cells of seven groups (n = 5). Data were presented as the mean ± SD. *P < 0.05; **P < 0.01; #P < 0.05; ##P < 0.01.

